# The Effects of Vector Movement and Distribution in a Mathematical Model of Dengue Transmission

**DOI:** 10.1371/journal.pone.0076044

**Published:** 2013-10-21

**Authors:** Dennis L. Chao, Ira M. Longini, M. Elizabeth Halloran

**Affiliations:** 1 Center for Statistics and Quantitative Infectious Diseases, Vaccine and Infectious Disease Division, Fred Hutchinson Cancer Research Center, Seattle, Washington, United States of America; 2 Department of Biostatistics, College of Public Health and Health Professions, and Emerging Pathogens Institute, University of Florida, Gainesville, Florida, United States of America; 3 Department of Biostatistics, School of Public Health, University of Washington, Seattle, Washington, United States of America; Northeastern University, United States of America

## Abstract

**Background:**

Mathematical models have been used to study the dynamics of infectious disease outbreaks and predict the effectiveness of potential mass vaccination campaigns. However, models depend on simplifying assumptions to be tractable, and the consequences of making such assumptions need to be studied. Two assumptions usually incorporated by mathematical models of vector-borne disease transmission is homogeneous mixing among the hosts and vectors and homogeneous distribution of the vectors.

**Methodology/Principal Findings:**

We explored the effects of mosquito movement and distribution in an individual-based model of dengue transmission in which humans and mosquitoes are explicitly represented in a spatial environment. We found that the limited flight range of the vector in the model greatly reduced its ability to transmit dengue among humans. A model that does not assume a limited flight range could yield similar attack rates when transmissibility of dengue was reduced by 39%. A model in which mosquitoes are distributed uniformly across locations behaves similarly to one in which the number of mosquitoes per location is drawn from an exponential distribution with a slightly higher mean number of mosquitoes per location. When the models with different assumptions were calibrated to have similar human infection attack rates, mass vaccination had nearly identical effects.

**Conclusions/Significance:**

Small changes in assumptions in a mathematical model of dengue transmission can greatly change its behavior, but estimates of the effectiveness of mass dengue vaccination are robust to some simplifying assumptions typically made in mathematical models of vector-borne disease.

## Introduction

Mathematical models of dengue transmission can help elucidate the dynamics of infectious disease transmission and will likely play a role in planning for interventions such as mass vaccination [1], [2]. Dengue is a vector-borne disease that infects an estimated 390 million individuals per year, resulting in about 96 million illnesses [3]. Because the outcome of interest is usually the infection of humans, a minimal mathematical model could represent the transmission of the dengue virus from infected to susceptible human hosts without explicitly representing the vector, *Aedes aegypti*. Most deterministic models include both the human hosts and the mosquito vectors, but the level of mechanistic detail can vary. One of the most common assumptions used in mathematical models of vector-borne disease transmission is that of homogeneous mixing of the vectors and their human hosts [1], [4]–[8]. In other words, all humans in the population are at equal risk of being infected by each mosquito and vice versa. At the other extreme, individual-based models can be used to explicitly represent the locations of each human and mosquito and infection can only occur between co-located hosts and vectors. However, more complicated models require more parameters and are more difficult to analyze.

Models of vector-borne disease have been studied for nearly a century, starting with the malaria model of Ross [4]. Dengue modeling is less mature [1], and there may be important differences between the two diseases important to modeling. Because the primary vector for dengue, *Aedes aegypti*, is a mosquito that flies short distances [9]–[11], a homogeneous mixing assumption might not be appropriate. Dengue cases tend to be clustered in both space and time [12]–[14], possibly a consequence of the short-range movements of *A. aegypti*. There are also complex immune interactions among the circulating serotypes, which could cause temporal and spatial patterns of infection [15]–[18].

Here, we use an individual-based model of dengue transmission to examine the effect of making different assumptions about mosquito movement and distribution. In this model, individual households are represented in space, and infectious mosquitoes can travel among the households, schools, and workplaces. The model also represents the four dengue serotypes, and infection by one serotype confers short-term protection against infection by the others. Using an individual-based model allows one to easily change assumptions about mosquito movement and spatial distribution. Because of recent progress towards producing a dengue vaccine [19]–[22], we study both the human infection rates and the effectiveness of mass vaccination in the models with differing assumptions.

## Methods

We used an individual-based model to simulate the spread of dengue in a semi-rural region in Thailand over a single year. A full description of this model, including the synthetic population and parameter settings, is in [23], and the model was run with exactly the same parameter values except where noted in the Results. The version of the model used in this work was downloaded from https://github.com/tjhladish/dengue/ on March 15, 2013. In brief, a synthetic population of 207,591 individuals with demography based on that of rural Thailand was assigned to households according to census data [24], and households were assigned continuous values for latitudes and longitudes to satisfy gridded population density estimates for the region [25]. Each day, these individuals travel between their households and workplaces or schools, as appropriate for their ages and using a gravity model to determine commuting distance for workers as described in Supporting Text S1 in [23].

**Figure 1:**
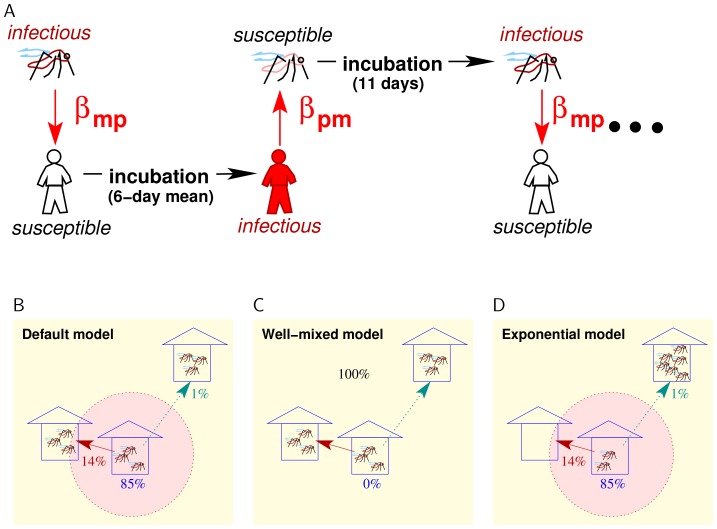
Mathematical model of dengue transmission. (A) In the model, infectious mosquitoes transmit dengue to susceptible humans with a probability of 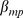 per bite. After an average incubation period of six days, humans transmit dengue to susceptible mosquitoes with a probability of 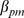 when bitten. Mosquitoes have an incubation period of eleven days, after which they are infectious. (B) In the *default* version of the model, mosquitoes travel to other locations with a probability of 15% per day (and stay in the same location with an 85% probability). Usually, they travel to nearby locations, but they can travel to randomly selected and arbitrarily distant location with a small probability. (C) In the *well-mixed* model, mosquitoes travel to a randomly selected location each day with essentially a 0% probability of remaining in the same location for two consecutive days. (D) In the *exponential* model, the number of mosquitoes per location is drawn from an exponential distribution instead of a uniform one, and mosquitoes move as in the default model.

All of these locations are associated with a mosquito population that can infect or be infected by humans. The model runs in one-day time steps, and the daily probability of contact between an individual human and a single mosquito is proportional to the amount of time they spend in the same location. The proportion of human–mosquito contact that occurs in the household is proportional to the amount of estimated mosquito biting activity before 9am and after 5pm, while the remaining transmission occurs in the locations where individuals are during the day, which may be work, school, or home (see Supporting Text S1 in [23]). For computational efficiency, we do not explicitly model the movement of susceptible mosquitoes because they do not affect the transmission of dengue (see Supporting Text S1 in [23] for implementation details). Infectious mosquitoes can transmit dengue to susceptible humans who are in the same location with a probability of 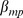 per mosquito bite, and infectious humans can transmit to susceptible mosquitoes with a probability of 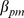 per bite (Figure 1A). Transmission probabilities were calibrated as described in Supporting Text S2 in [23]. Symptomatic individuals may stop going to work or school, and symptoms may begin one day before an infected person becomes infectious (see Supporting Text S1 of [23]). An individual mosquito tends to remain in a single location, but occasionally migrates to an adjacent location and can, rarely, travel to a randomly selected and possibly distant location (Figure 1B). Mosquitoes have a constant biting rate during their limited lifespan. Each location (i.e., household, classroom, workplace) can have the same number of mosquitoes or the number of mosquitoes at each location can be drawn from an exponential distribution.

There are two options for the mode of protection conferred by vaccination in the model: *all-or-none* and *leaky* [26]. For all-or-none protection, an individual is completely protected from infection by each of the four dengue serotypes with a probability of one minus the vaccine efficacy, with the probability of protection drawn independently for each of the four serotypes. For leaky protection, in which vaccinated individuals are x% less likely to become infected per infectious mosquito bite, where x% is one minus the vaccine efficacy.

The size of the mosquito population changes seasonally in the model, and the June peak in the mosquito population causes a peak in dengue cases in humans one or two months later. We do not model the mechanisms for the seasonal fluctuations in the mosquito population. Instead, the size of the mosquito population is reset each month to appropriate levels based on seasonal observations of adult mosquitoes to force the mosquito population size to follow realistic dynamics as described in Supporting Text S2 of [23].

Dengue is hyperendemic in the modeled region, so the level of exposure to all four serotypes is relatively high. Individuals in the synthetic population are assigned exposure histories to the four dengue serotypes based on their age and on estimates of dengue serotype prevalence in Thailand for the past forty years [23]. When running the model, a small fraction of individuals are continually exposed to dengue to prevent local stochastic extinction of a serotype. Because we assume that a vaccinated region would be adjacent to non-vaccinated hyperendemic regions, we do not think that local elimination of serotypes would be realistic. This assumption would not apply to geographically isolated populations or to non-hyperendemic areas. At the beginning of each simulated day, eight randomly selected individuals from the human population are exposed to dengue (two people to each of the four serotypes). This exposure accounts for both the introduction of dengue-infected humans and vectors and the exposure to dengue of residents who travel outside the model region. The magnitude of this influx affects the infection attack rate, particularly during the peak of dengue transmission from June to October. However, the model was calibrated to generate realistic annual infection attack rates using this arbitrary but low level of influx [23], and changes to this influx rate would produce qualitatively similar results after model re-calibration. If the person is not immune to the serotype, whether by prior exposure to the same serotype, cross-protection from recent infection by any serotype, or vaccination, that person becomes infected.

## Results

### The effect of mosquito movement and distribution assumptions on dengue incidence

**Figure 2:**
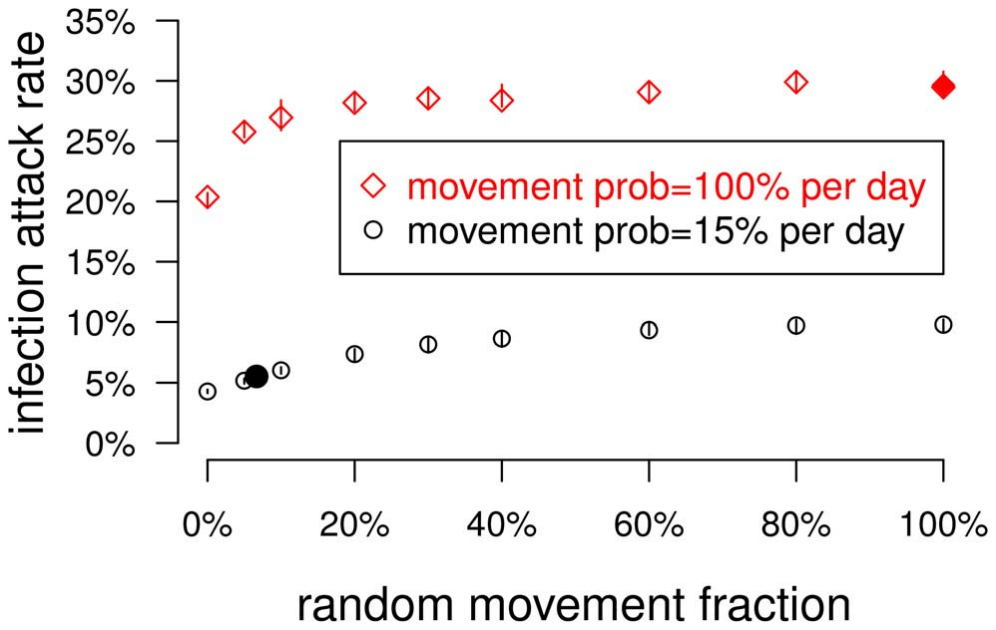
The effect of mosquito mobility on dengue transmission in the model. In the model, a parameter determined the probability that a mosquito's movement would take it to a neighboring location versus a random and possibly distant location. The plot shows the relationship between the infection attack rate among humans and this parameter. The lower points (black circles) plot attack rates when mosquitoes moved with a 15% probability per day, and the upper points (red diamonds) plot the attack rates when the mosquitoes moved every day. Each point represents the median of 40 stochastic simulations, and the vertical lines span the inter-quartile range. The two filled points highlight the attack rates corresponding to the default model in which mosquitoes tended to stay in one location (Figure 1B) and the well-mixed model in which mosquitoes moved to random locations each day (Figure 1C).

We studied the relationship between mosquito mobility and dengue transmission in the model. The *default* version of the model was calibrated to produce a 5.5% infection attack rate in the human population under the assumption that each mosquito moves with a 15% probability each day, usually to a neighboring location but with a 6.7% chance (i.e., a 1% probability per day) of moving to a randomly selected location regardless of distance (Figure 1B) [23]. We define the infection attack rate to be the total number of individuals infected by any of the four circulating serotypes during a simulated year divided by the population size. If mosquitoes were confined to moving only to neighboring locations, the attack rate was reduced to 4.3% (Figure 2 and Table 1). When the spatial restrictions on mosquito flights were eliminated and mosquitoes moved to any location with a 15% probability each day, the infection attack rate rose to nearly 10% (Figure 2). However, even in this model, mosquitoes tend to stay in the same location each day.

**Table 1:** Effect of mosquito movement and spatial distribution on human infection attack rates.

	pr move, %	random/short	*β_pm_*	*β_mp_*	mosq per loc, mean (distr)	AR, mean % ± std dev
*a*	15	6.7/93.3	0.100	0.250	42 (uniform)	5.5±0.5
	15	0/100	0.100	0.250	42 (uniform)	4.3±0.3
	15	100/0	0.100	0.250	42 (uniform)	9.8±0.8
	100	0/100	0.100	0.250	42 (uniform)	20.1±1
	100	1/99	0.100	0.250	42 (uniform)	22.9±1.4
	100	50/50	0.100	0.250	42 (uniform)	29.2±1.4
	100	100/0	0.100	0.250	42 (uniform)	29.7±1.2
*b*	100	100/0	0.079	0.1975	42 (uniform)	5.8±0.6
*c*	15	6.7/93.3	0.100	0.250	43 (exponential)	5.4±0.6

The parameters summarized are the daily probability that an individual mosquito moves to a new location, the ratio of moves that are random vs. short range, the transmission probability per infectious bite from person to mosquito (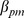), the transmission probability per infectious bite from mosquito to person (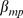), the mean number of mosquitoes per location and the distribution, and the mean and standard deviation of the human dengue attack rate (AR). Three scenarios are highlighted and are illustrated in Figures 1B–D: a) is the default, which includes mostly short-range but some random (i.e., not restricted by distance) mosquito movement and a uniform distribution of mosquitoes, b) is the well-mixed model, with frequent random mosquito movement and a uniform distribution of mosquitoes, and c) has the same mosquito movement as the default but the number of mosquitoes per location is drawn from an exponential distribution. The infection attack rates are reported as the means and standard deviations from 40 stochastic runs.

In a version of the model in which the mosquito population was *well-mixed*, mosquitoes moved to a random location each day regardless of flight distance (Figure 1C). In the well-mixed model, the infection attack rate was 30% (Table 1). If mosquitoes moved every day but were restricted to moving only to nearby locations, the infection attack rate was still high (about 20%) and attack rates rose quickly as the fraction of random movement increased (Figure 2). To reduce the well-mixed model's attack rate to a more realistic 5%, the transmissibility from person-to-mosquito (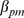) *and* from mosquito-to-person (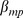) had to be reduced by 21%. In other words, dengue transmissibility needed to be reduced by about 38% to match the dengue incidence of the default model with spatially constrained mosquito movement. Mosquitoes are more likely to infect humans if they can immediately move to other locations, which would be likely to have more susceptible humans for at least two reasons: 1) The human who infected the mosquito is immune to that serotype, and 2) other mosquitoes infected by the same human are competing for susceptible human hosts in the same location. Infectious mosquitoes in the well-mixed model were more likely to infect humans than in the default model (19% vs 16%) and were more likely to cause infections in more than one location (46% vs 27%). We conclude that the vectorial capacity of mosquitoes is greatly reduced by their tendency to stay in a single building and to fly only short distances.

**Figure 3:**
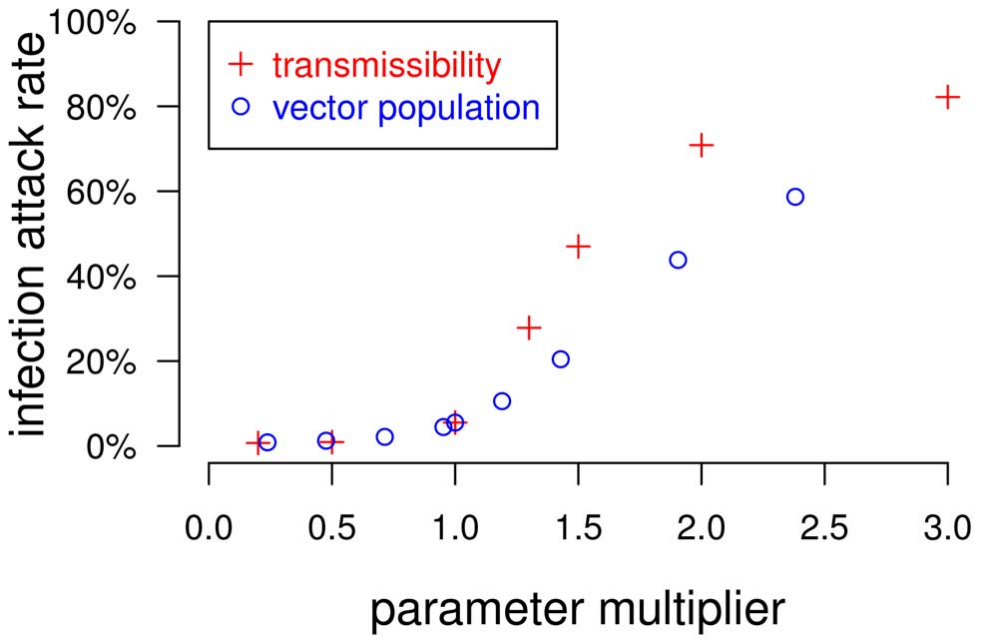
The sensitivity of the human infection attack rate to dengue transmissibility and the mosquito population size. Parameters for dengue transmissibility and the mosquito population size were varied with respect to the parameters of the default model. The red +s plot the average human infection attack rates from simulations in which the two transmission parameters, 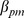 and 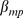, are set to values *x* times the default value, as indicated on the x-axis. The blue circles plot the results from simulations in which the mosquito population size is *x* times the default. The results from the two sets of models converge at x = 1.0, where all parameters match the default model's. Each point shows the average human dengue infection attack rate from 40 stochastic runs.

In the models described above, each location had the same number of resident mosquitoes. When the number of mosquitoes per location was drawn from an exponential distribution with the same mean number of mosquitoes per location (Figure 1D), transmission is reduced somewhat (Table 1). With an exponentially distributed number of mosquitoes per location, increasing the mean number of mosquitoes per location from 42 to 43 yields similar attack rates to the default model with uniformly distributed mosquitoes. We found the human infection attack rate to be more sensitive to transmissibility than to the mosquito population size (Figure 3).

### Simulating mass vaccination against dengue

**Figure 4:**
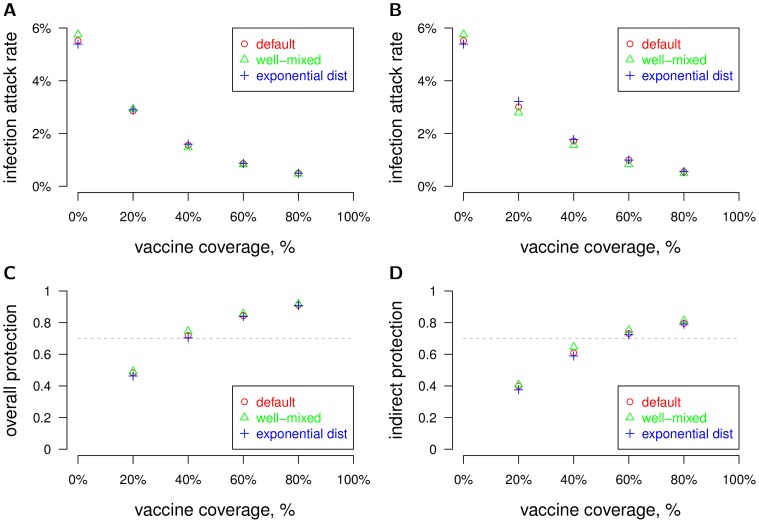
The effectiveness of mass vaccination under different mosquito movement and distribution assumptions. Mass vaccination of the population was simulated under different model assumptions of mosquito movement and distribution. We plotted the attack rate vs vaccine coverage of the human population for three different model variants, as shown in Figures 1B–D. The three models were calibrated to have similar attack rates with no vaccination (0% coverage). The parameters for these three models are summarized in Table 1. The mean attack rates from 40 stochastic runs per scenario are plotted. (A) Protection by vaccine is all-or-none (the default) with 70% efficacy. (B) Protection by vaccine is leaky with 70% efficacy. (C) Overall protection vs coverage is plotted. Vaccine efficacy is 70%, as indicated by the horizontal dashed line. (D) Indirect protection vs coverage is plotted.

We measured the effectiveness of mass vaccination of the human population in the models described above. The vaccine was 70% effective at preventing infection with any of the four dengue serotypes in the vaccinee. Therefore, 70% of vaccinees were completely protected against infection by a given dengue serotype. The probability of protection against a single serotype was independent of protection against the other three for a given vaccinated individual, so the probability that a vaccinated individual was protected against all four dengue serotypes was 70%^4^ = 24%. When the three models with different assumptions about mosquito movement and spatial distribution were calibrated to have the same attack rates in the absence of vaccination, mass vaccination had similar effects on human infection attack rates for all levels of coverage tested (Figure 4A). In the mass vaccination simulations, all individuals had the same probability of being vaccinated regardless of age. We also tested versions of the model in which protection conferred by the vaccine was *leaky* instead of *all-or-none*, so that vaccinees had a 70% reduction in susceptibility to infection *per infectious mosquito bite*. There was little difference in effectiveness of mass vaccination when the vaccine protection was leaky instead of all-or-none (Figure 4B). Overall protection of the vaccine, defined as one minus the attack rate within a partially vaccinated population divided by the attack rate within a totally unvaccinated population [27], surpassed 70%, the efficacy of the vaccine, once 40% of the population was vaccinated for all three versions of the model (Figure 4C). We found that *indirect* protection, defined as one minus the attack rate among unvaccinated individuals in a partially vaccinated population divided by the attack rate among a fully unvaccinated population, surpassed 70% once 60% of the population was vaccinated (Figure 4D).

## Discussion

Using an individual-based model of dengue transmission, we explored how different assumptions about the movement and spatial distribution of the vector can affect dengue incidence in humans. We found that the tendency for the most important vector of dengue, *A. aegypti*, to stay within a building and fly only short distances greatly affects its vectorial capacity. However, once a model is calibrated to obtain a realistic human infection attack rate, the population-wide effectiveness of mass dengue vaccination is robust to these assumptions. Although the model variants were calibrated to produce the same *average* infection attack rates, the spatial heterogeneity of infection risk could be different. When mosquito mobility is low, dengue risk would be elevated near dengue cases and lower elsewhere. In areas of high risk, a vaccine that confers “leaky” protection would be less effective in areas of high risk than one that confers “all-or-none” protection. However, the difference in the effectiveness of mass vaccination using the leaky or all-or-none assumption was minor.

Households may be the primary venue for dengue infection in humans. *A. aegypti* are known to breed in small containers associated with households, which may lead to spatial clustering at the household level [28]. Clusters of cases appear in households, consistent with a single source of infection [12], [13]. Households members of symptomatic dengue cases have been found to have a relatively high probability of being infected, though often asymptomatically [14]. These phenomena are best captured by mathematical models that explicitly include households, such as the model used here. In one model variant, we assumed an exponential instead of uniform distribution for the number of mosquitoes per location. Because there is little empirical data on adult mosquito counts per household, we chose a simple distribution that requires only one parameter. Using a more realistic or data-driven distribution might affect the results.

Dengue outbreaks often occur in small spatial clusters, probably because of the short flight range of *A. aegypti*. Perifocal spraying measures typically target a 100 meter radius around detected cases of dengue fever. Studies have found that the risk of dengue infection was significantly higher for children living within 100 meters of another infected child than in control clusters [29]–[31]. The elevated risk of infection in these clusters was for only a few days, evidence that risk was associated with individual dengue outbreaks [32]. The focal nature of dengue outbreaks can also be detected in spatial patterns of immunity to the four serotypes [18]. Therefore, spatial effects might play an important role in the multi-year dynamics of dengue.

A recent modeling study found that the fine-scale spatial distribution of mosquitoes could affect dengue transmission, since some houses had a superabundance of mosquitoes [33]. Our results indicate that such fine-scale heterogeneity might “wash out” when studying larger geographic areas. Including the ability for mosquitoes to disperse to other households dilutes the differences in mosquito populations in households with different production rates [34], [35]. We did not study coarser scales of spatial heterogeneity, such as regions that include both urban and rural populations.

Human movement probably plays a role in the spatial spread of dengue. A recent study demonstrated that visiting households with dengue-infected individuals was associated with an increased risk of infection [36]. Additional evidence for the role of human movement on the spread of dengue is the apparent spread of dengue along major roads [37]. In our model, we assumed that symptomatic people tend to stay home from school and work, which increases transmission in households. Thus, it may be important to capture these detailed human movements to estimate the risk of dengue, which was not our focus here.

We found that the incidence of dengue in the model was highly sensitive to the parameters associated with transmissibility but less so to the size of the mosquito population, which is consistent with simple deterministic compartmental models of vector-borne pathogens [4], [5]. Therefore, it may be unwise for modelers to borrow point estimates of transmission parameters directly from the experimental literature. It may also be unwise to extract parameter values from other models, since models with different assumptions may produce different results even with the same parameterization. For example, we found that when vectors in the model are not constrained by space, they were able to spread dengue much more effectively. Here, we decided to adjust transmissibility and vector population size in the different model variants in order to obtain consistent and realistic human infection attack rates.

Keeping the limitations of our mathematical model in mind, we can draw a few general conclusions. Although fine-grained spatial heterogeneity in the mosquito population likely has significant effects on dengue transmission within individual households, the ability of mosquitoes, and people, to move reduces the effects of these heterogeneities. Focal interventions, such as perifocal spraying, may require careful and fine-grained spatial modeling [33], but interventions that cover large regions, such as mass vaccination, might be insensitive to these features. However, there are features relevant to dengue transmission that occur at a coarser spatial scale, such as urbanization or mosquito habitat differences, and modeling dengue outbreaks in these regions probably requires a better understanding of dengue epidemiology.
